# Continuity of transcriptomes among colorectal cancer subtypes based on meta-analysis

**DOI:** 10.1186/s13059-018-1511-4

**Published:** 2018-09-25

**Authors:** Siyuan Ma, Shuji Ogino, Princy Parsana, Reiko Nishihara, Zhirong Qian, Jeanne Shen, Kosuke Mima, Yohei Masugi, Yin Cao, Jonathan A. Nowak, Kaori Shima, Yujin Hoshida, Edward L. Giovannucci, Manish K. Gala, Andrew T. Chan, Charles S. Fuchs, Giovanni Parmigiani, Curtis Huttenhower, Levi Waldron

**Affiliations:** 1 Department of Biostatistics, Harvard T.H. Chan School of Public Health, Boston, MA USA; 2 Department of Medical Oncology, Dana-Farber Cancer Institute, Boston, MA USA; 3 Department of Computer Science, Johns Hopkins University, Baltimore, MD USA; 4 Department of Pathology, University of Texas, Southwestern Medical Center, Dallas, TX USA; 5 Department of Nutrition, Harvard T.H. Chan School of Public Health, Boston, MA USA; 6 Department of Pathology, Brigham and Women’s Hospital, Boston, MA USA; 7 Gastroenterology, Department of Medicine, Massachusetts General Hospital, Boston, MA USA; 8 Department of Biostatistics and Computational Biology, Dana-Farber Cancer Institute, Boston, MA USA; 9 Graduate School of Public Health and Health Policy, City University of New York, 55 W 125th St, New York, NY 10027 USA; 10 Institute of Implementation Science in Population Health, City University of New York, New York, NY USA

**Keywords:** Colon cancer, Tumor, Transcriptional profiling, Progression

## Abstract

**Background:**

Previous approaches to defining subtypes of colorectal carcinoma (CRC) and other cancers based on transcriptomes have assumed the existence of discrete subtypes. We analyze gene expression patterns of colorectal tumors from a large number of patients to test this assumption and propose an approach to identify potentially a continuum of subtypes that are present across independent studies and cohorts.

**Results:**

We examine the assumption of discrete CRC subtypes by integrating 18 published gene expression datasets and > 3700 patients, and contrary to previous reports, find no evidence to support the existence of discrete transcriptional subtypes. Using a meta-analysis approach to identify co-expression patterns present in multiple datasets, we identify and define robust, continuously varying subtype scores to represent CRC transcriptomes. The subtype scores are consistent with established subtypes (including microsatellite instability and previously proposed discrete transcriptome subtypes), but better represent overall transcriptional activity than do discrete subtypes. The scores are also better predictors of tumor location, stage, grade, and times of disease-free survival than discrete subtypes. Gene set enrichment analysis reveals that the subtype scores characterize T-cell function, inflammation response, and cyclin-dependent kinase regulation of DNA replication.

**Conclusions:**

We find no evidence to support discrete subtypes of the CRC transcriptome and instead propose two validated scores to better characterize a continuity of CRC transcriptomes.

**Electronic supplementary material:**

The online version of this article (10.1186/s13059-018-1511-4) contains supplementary material, which is available to authorized users.

## Background

Several sub-classification systems of colorectal carcinoma (CRC) have been developed, defined by genomic or epigenomic features (chromosomal instability, microsatellite instability, and CpG island methylator phenotype), alterations of a single driver gene (such as *KRAS*, *BRAF*, etc.), or a combination thereof [1–4]. Recently, progress has also been made towards a transcriptome-based CRC classification system [5–7], as has been well established for breast carcinoma [8, 9]. A key advantage of such a system is that it reflects the downstream effects of genomic and epigenomic changes. Most prominent among these efforts, the CRC Subtyping Consortium in 2015 synthesized findings from previously published independent CRC classification studies and reported four concordant CRC subtypes (the CRC Consensus Molecular Subtypes [CMS1–4]) [10]. Alternatively, in 2017 Isella et al. reported five CRC “intrinsic” subtypes (CRIS) that are more robust against stromal confounding in the tumor transcriptome [11]. In all these works, the authors characterized CRC tumor subtypes with their implications in terms of molecular (e.g. microsatellite instability), histopathological (e.g. tumor stage), and clinical (e.g. survival outcome) variables. However, given that gene expression and tumor phenotype are variably influenced by external environment and endogenous factors [12], we hypothesized that the biological diversity of CRC may be better represented by a continuum of reproducible variations rather than by discrete subtypes.

To test our hypothesis, we conducted a series of meta-analyses [13] incorporating 18 published CRC transcriptome datasets. We adopted an established quantitative evaluation framework utilized in previous literature [14–16] to study the discreteness of previously published subtypes in the CRC transcriptome [10, 11]. Specifically, we applied different clustering strength metrics [17–19], which quantitatively evaluate whether the transcriptomes of different CRC subtypes form discrete “clusters.” These measures assess the similarity of expression profiles in the same cluster compared to those in different clusters [17], compare within-cluster dispersion to a reference null distribution [18], and assess robustness to re-training the classifier in new datasets [19]. We applied these metrics to previously proposed subtype classifiers [10, 11] and to de novo subtypes [20–22] across numerous datasets including a stroma-filtered dataset. We found that a set of continuously variable, reproducible gene expression patterns agree with and subsume previously proposed discrete subtypes and can be more robustly replicated in validation studies. The proposed “continuous subtypes” or scores offer a novel, precise characterization of CRC tumors that allows for better-powered therapeutic effect evaluation and individualized treatment assignment.

## Results

### Discreteness of CRC transcriptional subtypes cannot be validated

**Fig. 1:**
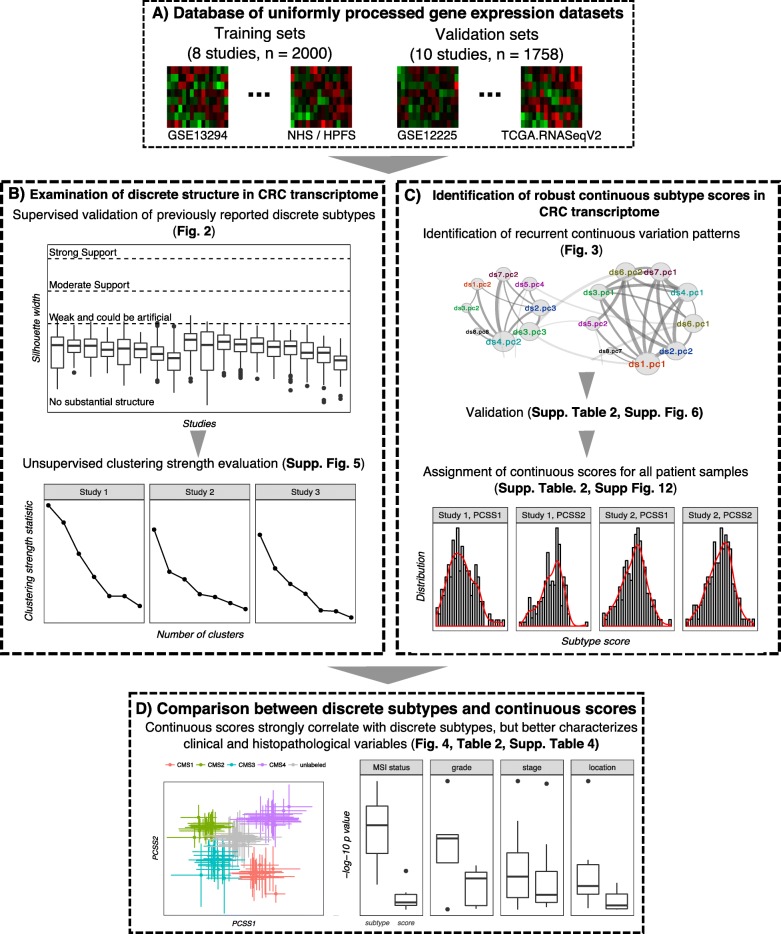
Overview of analyses performed in this study. Shown here are the steps carried out to examine the validity of discrete subtypes, as well as to identify, validate, and characterize continuously variable subtypes for CRC transcriptomes

We first examined the robustness of discrete CRC transcriptional subtypes, and in particular the CMS1–4 by the CRC Subtyping Consortium [10] (hereafter referred to as the Consortium), on a collection of 18 published studies (Table 1, Fig. 1). Among all past CRC transcriptome subtyping efforts, we prioritized the Consortium’s CMS results. This is because they represent concordant subtypes across multiple independent transcriptional classification systems and are, to date, still the most comprehensive, well-powered, and well-validated classification study [23, 24]. For the Consortium and other previous transcriptional subtyping efforts, an important assumption is that clear distinctions exist in the transcriptomes of the determined CRC subtypes (i.e. they are separable and “discrete”). We tested the validity of this hypothesis in each of the 18 published studies via: (1) supervised validation of separation between the Consortium subtypes with a widely adopted quantitative framework; and (2) complementary and de novo unsupervised clustering analysis and cluster strength evaluation.

**Table 1:** Clinical characteristics of selected training and validation sets used in this study

Dataset	Accession ID	Platform	Tumor / Normal samples (n)	Late stage tumors (%)	Staging system	Availability of metastasis info
Training sets						
Jorissen and Sieber, 2008b [53]	GSE13294	[HG-U133_Plus_2] Affymetrix Human Genome U133 Plus 2.0 Array	155/0	–	–	No
Watanabe and Hashimoto, 2008 [54]	GSE14095	[HG-U133_Plus_2] Affymetrix Human Genome U133 Plus 2.0 Array	189/0	–	–	No
Jorissen and Sieber, 2008 [55]	GSE14333	[HG-U133_Plus_2] Affymetrix Human Genome U133 Plus 2.0 Array	290/0	77.55	TNM/Duke	Yes
Smith and Beauchamp, 2009a [56]	GSE17536	[HG-U133_Plus_2] Affymetrix Human Genome U133 Plus 2.0 Array	177/0	80	TNM/Duke	Yes
Mori, Mimori, Yokobori T, 2010 [57]	GSE21815	Agilent-014850 Whole Human Genome Microarray 4x44K G4112F (Probe Name version)	131/9	59.54	TNM/Duke	Yes
Vilar and Morgan, 2011a [58]	GSE26682.GPL570	[HG-U133_Plus_2] Affymetrix Human Genome U133 Plus 2.0 Array	176/0	–	–	No
Vilar and Morgan, 2011b [58]	GSE26682.GPL96	[HG-U133A] Affymetrix Human Genome U133A Array	155/0	–	–	No
NHS-HPFS [41]	GSE32651	Illumina DASL HumanRef-8 v3	718/0	13.83	TNM	No
Validation sets						
Lips and Morreau, 2008 [59]	GSE12225.GPL3676	NKI-CMF *Homo sapiens* 35 k oligo array	42/0	28.57	TNM	Yes
Staub and Rosenthal, 2009 [60]	GSE12945	[HG-U133A] Affymetrix Human Genome U133A Array	62/0	41.94	TNM	Yes
Jorissen and Sieber, 2008a [53]	GSE13067	[HG-U133_Plus_2] Affymetrix Human Genome U133 Plus 2.0 Array	33/0	–	–	No
Smith and Beauchamp, 2009b [56]	GSE17538.GPL570	[HG-U133_Plus_2] Affymetrix Human Genome U133 Plus 2.0 Array	63/0	88.1	TNM/Duke	Yes
expO, IGC, 2005	GSE2109	[HG-U133_Plus_2] Affymetrix Human Genome U133 Plus 2.0 Array	427/0	51.6	TNM/Duke	Yes
Tsukamoto and Sugihara, 2010 [61]	GSE21510	[HG-U133_Plus_2] Affymetrix Human Genome U133 Plus 2.0 Array	123/25	79.57	TNM/Duke	Yes
Medema and Tanis, 2011 [62]	GSE33113	[HG-U133_Plus_2] Affymetrix Human Genome U133 Plus 2.0 Array	90/6	–	TNM/Duke	Yes
Marisa and Boige, 2012 [63]	GSE39582	[HG-U133_Plus_2] Affymetrix Human Genome U133 Plus 2.0 Array	566/0	87.75	TNM/Duke	Yes
TCGAa [5]	TCGA.COAD	Agilent 244 K Custom Gene Expression G4502A-07-3	122/4	42.4	TNM	Yes
TCGAb [5]	TCGA.RNASeqV2	[RNASeqV2] Illumina HiSeq RNA sequencing	181/14	53.09	TNM	Yes

The normal samples in these datasets were all from adjacent normal tissues. The percentage of late-stage and high-grade samples were calculated where the information is available

**Fig. 2:**
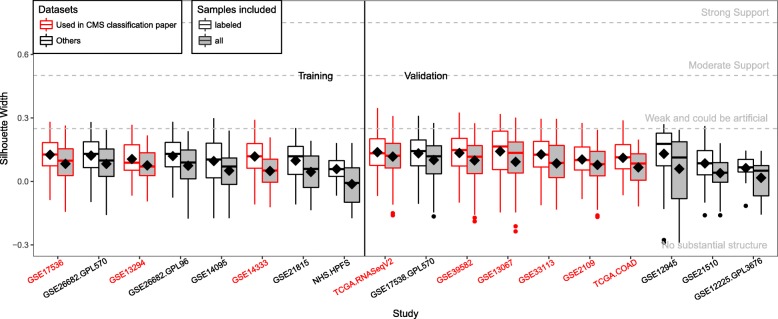
Previously published CRC subtypes do not separate samples’ transcriptional profiles. Average silhouette widths between the previously reported CMS [10] subtypes provide no evidence for substantial clustering structure. Silhouette widths for separation between CMS subtypes are calculated within each of the 18 datasets used in our study, either for all samples or only for those confidently labeled by the CMS classifier as provided in [10]. Distribution of samples’ silhouette widths is represented with *box plots*, with diamonds marking the average. Studies are separated according to training and validation sets, and then ranked based on their average silhouette widths. Datasets also used in the CMS paper are marked in *red*. The reference levels of clustering (*horizontal gray dashed lines*) are the same as in [25]. These results are not sensitive to dissimilarity measures (Additional file 1: Figure S1B)

Using an established evaluation framework in cancer transcriptional subtyping [14–16], we quantitatively evaluated separation between the Consortium subtypes, CMS1–4 (assigned in our datasets by the classifier provided by the authors, see “Methods” for details), using average silhouette width. Silhouette width is a statistic commonly adopted to summarize the level of separation between groups of samples and strength of clustering structure in the data [17]. This analysis is supervised in the sense that the class labeled are pre-defined by the CMS classifier. Average silhouette widths of CMS subtypes were < 0.25 in all of the 18 datasets (Fig. 2, Additional file 1: Figure S1), not exceeding the “no substantial clustering” threshold defined in previous literature [25], providing evidence that little separation exists between the CMS subtypes. The strength of separation decreases even more if we include samples that cannot be confidently classified into any of the four CMS subtypes, suggesting such samples form the “intermediate” group in the continuous distribution of different subtypes, as also noticed by the Consortium authors [10]. The lack of separation between CMS subtypes is visually noticeable in the distribution of top principal components (PCs) of each study (Additional file 1: Figure S2). This is in contrast to breast tumors, where separation between well-established transcriptional subtypes is prominent even in only the first two PCs (Additional file 1: Figure S3). These results suggest that CRC transcriptomes are distributed more as a continuum than discrete classes. Notably, nine of these 18 datasets were also used by the Consortium to validate the robustness of the CMS subtypes.

To rule out the possibility that lack of separation between subtypes is due only to lack of generalizability of the CMS classifier applied to new datasets, we performed de novo unsupervised clustering analysis with different algorithms (k-medoid [20], non-negative matrix factorization [21], and consensus clustering [22]) in each study. We evaluated discreteness of the resulting clusters by gap statistic [18], prediction strength [19], and average silhouette width [17]. De novo clusters (subtypes) also showed no evidence of discreteness or of preference for four clusters (the number of CMS subtypes), consistently across clustering algorithms and separation strength measures (Additional file 1: Figure S4). We thus conclude that the absence of discreteness in the CRC transcriptome is consistent, when investigated by a variety of methodologies and datasets.

### Continuous subtypes: common patterns of population-level transcriptional variation reproduced in multiple studies

We identified and validated “continuous subtypes” of the CRC transcriptome and showed that they are consistent with previously proposed discrete subtypes but offer better representation of tumor-to-tumor transcriptional variability. The continuous subtypes are characterized by patterns of variation between CRC patients that are consistent across multiple studies. They parallel the common paradigm of discrete molecular subtypes of cancer transcriptional activity, but an individual is represented by numerical scores rather than one of several discrete classes.

We developed a meta-analytical adaptation of principal component analysis (PCA) to identify consistent continuous scores across different studies, in the presence of cohort differences and potential study-specific batch effects (Additional file 1: Figure S5). Briefly summarized, we performed PCA on eight training datasets (Fig. 1, Table 1, Additional file 2: Table S1) and constructed a network of connected top PCs from all eight datasets to find non-study-specific major transcriptional shifts. PCs of different datasets were considered correlated and connected in the network if their corresponding loading vectors had an absolute Pearson correlation of > 0.5; this is interpreted as the recurrence of similar major transcriptional shifts in both studies.

**Fig. 3:**
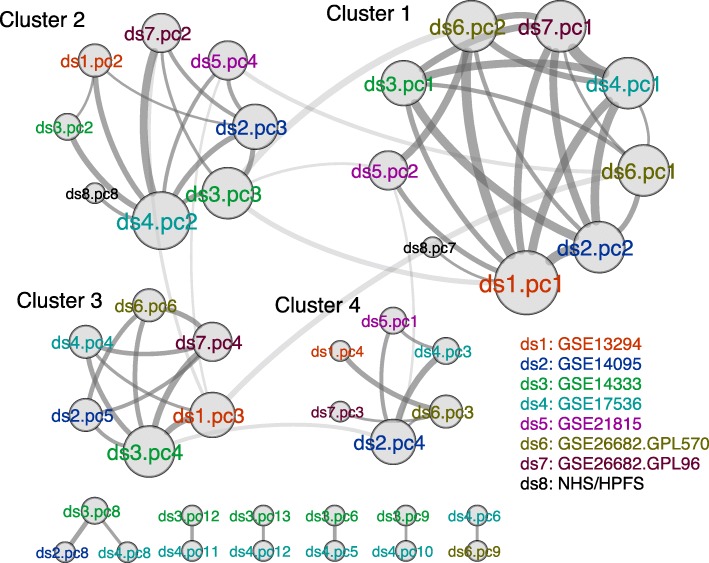
Correlated PCs from training datasets form densely connected clusters, characterizing robust major transcriptional shifts. These can then be used as basis for continuous subtype scores. Each node represents one of the top 20 PCs in one dataset (ds). Edges indicate an absolute Pearson correlation of at least 0.5 between the corresponding loading vectors (singletons are not included in the figure). Node size is proportional to its degree (the number of PCs that it is correlated with), and edge width is proportional to Pearson correlation. Clusters were identified based on the Girvan-Newman algorithm [49], which separated four large clusters, each corresponding to a recurrent “spectrum” of subtype scores (i.e. a pattern of coordinated gene expression differential across subjects within a dataset and recurring in multiple datasets). For the first seven training datasets, the PCs present in the four clusters were all top PCs, which means that the strongest signals for these datasets are all true signals. For the NHS/HPFS dataset, however, PCs 1–6 were missing. This suggests a strong batch effect (noise) in this particular dataset

In this network, we found four large clusters of densely interconnected PCs (Fig. 3). PCs from the same cluster characterize major transcriptional shifts of similar direction which were robustly observed across multiple datasets, independent of batch effects [26]. Within the clusters, PCs of different studies were of different ranks, reflecting varying levels of study-specific technical or biological effect. As an example, dataset 8 (NHS/HPFS, the only study performed on formalin-fixed, paraffin-embedded specimens) only had its seventh and eighth PCs correlated with other datasets, suggesting that its top six major transcriptional shifts did not recur in the rest of the training datasets and were likely study-specific.

Each PC cluster yielded a different way of assigning continuous scores to tumors, defined by the average of the loading vectors of that cluster (Additional file 3: Table S2, see “Continuous subtype discovery” in the “Methods” section for details on assigning the scores). The scores were, by definition, repeatedly observed across the training studies, because it was highly correlated with all datasets / PCs in the cluster. Once further validated, this score can be viewed as consistently describing a major direction of transcriptional variation and is used instead of discrete classes to characterize different CRC transcriptomes. That is, one can assign subtype scores, instead of specific subtypes, to different tumors. As shown in the validation results, clusters 1 and 2 were best validated in the additional 10 studies and we focus on these two clusters to provide robust characterization of CRC.

### Validation of subtype scores as major transcriptional shifts

The major transcriptional shifts identified by our method are highly reproducible across validation datasets, especially for clusters 1 and 2 (Additional file 4: Table S3). Using the same criteria as in the training stage, the average loading vectors of clusters 1 and 2 were both correlated (absolute Pearson correlation > 0.5) with top PCs in 9/10 validation datasets. In contrast, they were not correlated with top PCs of normal tissues-only datasets, and even less so with randomly selected PCs, or randomized datasets formed by permuting gene expressions (Additional file 1: Figure S6). Because of the strong replicability of these two average loading vectors, we termed the scores assigned by them PC Cluster Subtype Scores 1 and 2 (PCSS1 and PCSS2) and used these subsequently in place of discrete classes to characterize CRC tumors.

Similar to the idea of signature gene lists for discrete subtypes, from the continuous scores we also generated “signature” gene subsets with sizes of ~ 200 that can be used to sufficiently approximate the continuous scores (Additional file 1: Figure S7). In diagnostic or prognostic practice, these gene subsets can be used to robustly assign continuous subtype scores in place of the entire transcriptome.

### CRC subtype scores are continuous but still in agreement with previously established subtypes

We compared subtype scores PCSS1 and PCSS2 first against microsatellite instability (MSI), a well-established CRC subtype with distinct carcinogenic pathway [1], then with the CMS subtypes proposed by the Consortium. With meta-analyses [13] using fixed effects models, we found that tumor sample microsatellite instability is consistently correlated with higher PCSS1 scores (*p* = 1 × 10^−31^) and lower PCSS2 scores (*p* = 5 × 10^−71^) (Table 2, Additional file 5: Table S4). Although tumors are commonly categorized as microsatellite stable or unstable, the continuity in the distribution of the subtype scores suggests no discrete separation of MSI patients, as quantitatively evidenced by silhouette width (Additional file 1: Figure S8). This is consistent with previous observations that all CRCs show some level of microsatellite instability [27] and indicates that MSI CRC can be identified by subtype scores.

**Table 2:** Estimated overall effect size and *p* values for continuous scores on molecular, histopathological, and clinical variables from fixed effects model

Variable	Continuous score	Effect size	*p* value
CMS1 subtype	PCSS1	0.82	3E-55
	PCSS2	− 2.55	1E-129
CMS2 subtype	PCSS1	− 2.00	2E-156
	PCSS2	0.76	7E-60
CMS3 subtype	PCSS1	− 0.57	2E-28
	PCSS2	− 0.75	6E-56
CMS4 subtype	PCSS1	1.72	2E-130
	PCSS2	1.75	4E-106
MSI	PCSS1	0.76	1E-31
	PCSS2	− 1.68	5E-71
Right location	PCSS1	0.087	0.09
	PCSS2	− 0.23	1E-04
Late stage	PCSS1	0.16	0.002
	PCSS2	0.24	6E-06
High grade	PCSS1	0.33	3E-05
	PCSS2	− 0.30	7E-05
Disease recurrence or death	PCSS1	0.23	5E-05
	PCSS2	0.19	0.001

The effect size statistic is log hazard ratio for disease recurrence or death and log odds ratio for all other variables. These estimates are not sensitive to fixed vs random effects modeling (Additional file 5: Table S4). Statistics for individual datasets, including *I*^2^ statistics, are also provided in Additional file 5: Table S4

**Fig. 4:**
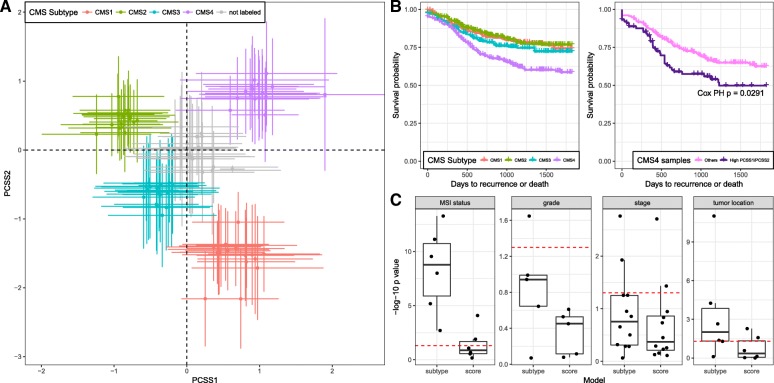
Continuous subtype scores consistently reproduce CMS subtypes, but provide additional information in characterizing molecular/histopathological/clinical correlates. **a** Subtype- and study-specific mean PCSS1 and PCSS2 scores indicate “quadrants” in the distribution of the continuous scores that correspond to CMS1–4 subtypes, with unlabeled samples clustered at the origin. Each point indicates the average PCSS1 and PCSS2 value of samples classified as a particular CMS subtype in one dataset, with *error bars* representing standard deviation. **b** CMS4 subtype has the worst DFS outcome in all samples where survival information is available, agreeing with results in [10], but stratification of CMS4 samples with respect to continuous scores reveals an even more highly at-risk subgroup at the extreme end of PCSS1/PCSS2 distributions. Individual hazard ratios for each study are included in Additional file 1: Figure S9C). Continuous scores are more closely associated with molecular and clinical/pathological variables than discrete subtypes. Molecular, histopathological, and clinical variables were regressed on subtypes and scores as covariates. LRTs were used to compare the full model, containing both subtype and score as predictors, to a simplified model containing only subtype (*left*) or score (right) as predictor. Test results for different datasets (*p* values) are represented by points in the box plots. A *p* value near 1 (−log-10 *p* value near 0) suggests that no additional information is provided by the full model, whereas a small *p* value suggests that the full model provides additional information for predicting molecular/clinical variables. The more significant *p* values for models using only discrete subtypes (*left*) vs continuous scores (*right*) suggest that discrete subtypes alone lack information provided by the full model; conversely, log-10 *p* values near zero for scores (*right*) suggest that continuous scores outperform discrete subtypes in characterizing the molecular and clinical/pathological variables

For the Consortium subtypes, distribution of the continuous scores form “quadrants” that correspond to each one of the four CMS subtypes. Specifically, CMS1 has high PCSS1 and low PCSS2, CMS2 has low PCSS1 and high PCSS2, CMS3 has low PCSS1 and PCSS2, and CMS4 has high PCSS1 and PCSS2 (Fig. 4a, Table 2, Additional file 5: Table S4). As expected, the “not labeled” samples, i.e. samples that cannot be confidently assigned by the Consortium classifier to any of the four subtypes, are distributed between the classified subtypes in the continuous score space. This again confirms that such samples are not transcriptionally distinct from those with subtype assignment, but simply are the intermediate samples in the continuous distribution of CRC transcriptomes. The two continuous scores encompass discrete subtypes, in the sense that together they capture the difference between tumors described by discrete subtypes and provide additional resolution in differentiating tumors in the form of numerical scores.

### Continuous subtypes correlate with location, stage, grade, and prognosis better than discrete subtypes

Besides MSI, PCSS1 and PCSS2 correlated with tumor location, stage, grade, and prognosis when fitting fixed effects models on all datasets with available information (Table 2, Additional file 5: Table S4). Specifically, right-sided tumor location was correlated with lower PCSS2 scores (*p* = 1 × 10^−4^). Because the correlation is in the same direction as MSI, they are consistent with previous observations that right-sided tumors tend to be MSI [28]. Late tumor stage was found to be significantly associated with higher PCSS1 and PCSS2 (*p* = 0.002 and 6 × 10^−6^, respectively), and high tumor grade with higher PCSS1 and lower PCSS2 (*p* = 3 × 10^−5^ and 7 × 10^−5^, respectively). High PCSS1 and PCSS2 were also associated with worse disease-free survival (DFS) outcome (*p* = 5 × 10^−5^ and 0.001, respectively). The above correlations agree with the subtype-clinical phenotype correlations reported by the Consortium [10]. For example, the CMS4 subtype was reported to have the worst prognosis, whereas in our study, high PCSS1 and PCSS2 are correlated with both CMS4 and worse DFS.

The continuous scores furthermore provide better capability in differentiating and explaining samples’ molecular, histopathological, and clinical characteristics, because they encompass previously proposed discrete subtypes. Using DFS again as an example: while the CMS4 subtype was reported to have the worst DFS outcome, further differentiating within the subtype using continuous scores (PCSS1 or PCSS2 greater than the upper quartile) identifies a subgroup with even higher risk (Cox proportional hazard regression *p* = 0.029, Fig. 4b, Additional file 1: Figure S9), capturing additional prognosis heterogeneity within the subtype. To statistically test for this or other binary variables, we performed likelihood ratio tests (LRT) in each dataset on regression models with MSI status, location, stage, or grade as the outcome. Covariates were chosen to be either the continuous subtype scores or discrete CMS subtypes, or both combined as a reference full model. LRT was performed between the reference full model and either the continuous scores only model or the discrete subtypes only model (see “Methods” for details). For all outcome variables, the model with only continuous scores has much larger (less significant) *p* values in the LRT, and lower Akaike information criterion (AIC) [29] than the discrete subtypes-only model (Fig. 4c, Additional file 5: Table S4). This suggests that continuous scores outperform discrete subtypes in characterizing samples’ molecular, histopathological, and clinical characteristics; heterogeneity in such variables among CRC tumors is better explained by continuous scores than by discrete subtypes.

### Continuous subtypes are enriched for inflammation and T-cell response pathways

We used pre-ranked gene set enrichment analysis to examine pathways associated with PCSS1 and PCSS2 (Additional file 6: Table S5), providing functional interpretations for the continuous scores. Specifically, we looked for pathways that were enriched for genes with large weights in the loadings of PCSS1 and PCSS2. Eight out of 217 Biocarta pathways are significantly enriched for important genes of PCSS1 (Bonferroni corrected *p* < 0.05). These pathways are associated with either T-cell functionality (TCRA, TCYTOTOXIC, THELPER, DC), and/or the inflammatory response (IL17, LYM, INFLAM, LAIR). Only the CDK Regulation of DNA Replication pathway is enriched among heavily weighted genes of PCSS2. PCSS1 and PCSS2 characterize the variation of these pathways.

### CRC subtype discreteness is not sensitive to tumor microenvironment heterogeneity

Tumor stromal content has been noted as a source of transcriptional variability that might affect CRC classification [30, 31]. Isella et al. [11] published new CRIS that are trained on xenograft tissues (GSE76402). Because they are derived exclusively on epithelial cells, they are reported to be better conserved across different tumor stromal content than the consensus CMS subtypes. Other evidence, however, shows that CMS subtypes are also well-conserved independently of stromal contribution [24]. We performed additional analysis to: (1) investigate the confounding effect of stroma contribution in the discreteness of CRC transcriptional subtypes; and (2) compare the proposed continuous scores to the stroma-free CRIS subtypes.

First, we find that there is also no evidence for discreteness of the CRIS subtypes, and specifically in stroma-filtered CRC transcriptomes. We assessed the discreteness of the intrinsic CRIS subtypes using only the gene set filtered for stromal signal as provided in [11], in the xenograft study GSE76402 where they are derived, in an independent CRC cell line dataset (GSE59857 [32]), and in all 18 bulk tissue studies. We applied the same supervised and unsupervised frameworks used to evaluate the CMS subtypes. As we show in Additional file 1: Figures S1 and S4, the discreteness measures provide no evidence for discreteness in a stroma-filtered transcriptome, including the xenograft and cell line datasets.

Second, PCSS1 and PCSS2 have consistent correlation with CRC CRIS subtypes in both regular cancer tissues and stroma-free samples, but better characterize the distribution of clinical, histopathological, and molecular variables. We performed linear regression between CRIS subtypes and PCSS1/PCSS2; the correlations are consistent not only in the 18 CRC bulk tissue studies, but also, importantly, in the GSE76402 xenograft and the GSE59857 cell line study (Additional file 1: Figure S10, Additional file 7: Table S6). Furthermore, we find that the continuous scores represent the distribution of cancer stage, grade, location, and MSI status better than CRIS subtypes, using the same likelihood ratio testing framework as applied to the CMS subtypes (Additional file 1: Figure S11). We thus conclude that lack of discreteness of the CRC transcriptome cannot be explained by stromal contamination.

## Discussion

We propose continuous scores that reflect the molecular epidemiology and population heterogeneity of colorectal cancer better than previously proposed discrete subtypes. These continuous scores were identified and validated across multiple independent datasets using a novel approach to unsupervised subtyping that does not assume the existence of discrete subtypes. In molecular classification of cancer subtype, discreteness tends to be assumed a priori (for example, ovarian [33] and cutaneous [34] carcinomas), despite having been questioned for both CRC and other types of tumor [35, 36]. We argue that in the case of colorectal cancer continuous subtype scores provide a more consistent description of transcriptional variation in CRC. Given the notion that each cancer is different [37, 38], we suggest that in future analysis either strong biological insight or careful validation be provided to justify the use of discrete subtypes.

Our proposed continuous scores for CRC, PCSS1 and PCSS2, are consistent with previously published discrete subtypes [10], generalize their expression patterns and associations to location, stage, grade, and DFS, and are observed consistently in validation datasets. The consistency with discrete subtypes is notable because the proposed continuous scores were trained on different datasets using different methodology. It is also worth noting that our approach did not assume that the subtypes in CRC transcriptome are necessarily continuous. The existence of strong, discrete subtypes is not supported by unsupervised clustering strength metrics (Additional file 1: Figure S4) or by visual and quantitative inspection of the proposed continuous scores, which do not show evidence of multimodal distribution (Additional file 1: Figure S12). In supervised investigation of published discrete subtypes, we also found little evidence of subtype discreteness in validation datasets through PCA visualization and quantitative silhouette width evaluation (Fig. 2, Additional file 1: Figure S1).

The most well-defined CRC molecular subtypes are chromosomal instability (CIN), MSI, and the CpG island methylator phenotype (CIMP) [1]. The transcriptome-based continuous subtype scores proposed here are strongly correlated to MSI: tumors with average PCSS1/2 can be either MSI or MSS, but MSI tumors only rarely having a distinctly MSS-like continuous subtype score. Functionally, MSI tumors involve a unique carcinogenic process that encompasses mutations in the coding mononucleotide repeats in tumor suppressor genes [1]. The distinction becomes less obvious in terms of gene expression phenotypes, however: as is observable from the continuity in the subtype scores despite their strong correlation with MSI and varying degree of MSI prevalence in CRC [27]. The association of continuous scores with CIN or CIMP subtypes in these data could not be tested here due to metadata availability but can be deduced based on the strong correlation between the scores and the Consortium subtypes, and the reported CIN and CIMP characteristic of each CMS subtype [10].

Continuous scores can be applied in practice as effectively as discrete subtypes and may be more appropriate for treatment targeting, risk assessment, and underlying molecular biology. For example, high PCSS1 and high PCSS2 are both associated with worse DFS (Table 2), suggesting that patients with such characteristics could be specifically targeted for more aggressive therapeutic regimens. Continuous variability in molecular phenotypes can easily be incorporated as features in survival or risk models, providing a stronger predictor of disease prognosis or outcome (Fig. 4b, Additional file 1: Figure S9). The scores could be obtained using a smaller set of representative genes, with a continuous tradeoff between reducing the number of genes measured and maintenance of the score obtained from a whole-transcriptome assay (Additional file 1: Figure S7). Employing improved models of tumor transcriptional activity that more closely reflect the underlying biological variability of the disease, such as those presented in this paper, should help improve the translation of genomic features into clinical practice.

## Conclusions

We examined subtype discreteness in the CRC transcriptome, a common but unvalidated assumption, and found consistent evidence suggesting lack of such patterns. We instead propose a novel method for identifying continuous subtype scores that are consistent across numerous independent datasets, which we applied to identify two PCA-based scores (PCSS1 and PCSS2) and to provide a gene signature that can be used in practice to obtain the proposed subtype scores. These are consistent with previous discrete subtypes in associations with clinical variables, including DFS, but better represent tumor-to-tumor CRC transcriptional variation and enable improved characterization of other molecular, histopathological, and clinical variables. These results are confirmed in stroma-filtered CRC cells. Continuous scores thus have the potential to differentiate patient subgroups, such as those with poor DFS, with greater personalization and precision than discrete subtypes.

## Methods

### Publicly available datasets

We based our analyses primarily on a collection of publicly available transcriptional studies on colorectal cancer (Fig. 1), as available in the curatedCRCData package [39] in Bioconductor. The package provides a total of 33 uniformly prepared gene expression data on CRC with documented and curated clinical metadata. Known technical replicates within the same study were merged as part of the curatedCRCData pipeline by taking the average, whereas unknown replicates across studies were identified with the doppelgangR package [40] and removed. We then selected seven training and 10 validation sets from the remaining samples of the 33 datasets (Table 1), based on the following inclusion criteria:

Inclusion criteria for training sets:

1. Only contains primary tumor samples
2. Sample size > 100
3. Affymetrix platform
4. Study was published after 2007

Inclusion criteria for validation sets:

1. Contains either primary tumors or primary tumors / normal control samples
2. Sample size > 60
3. Genes overlap with at least 90% of the common genes in the training sets.

The NHS/HPFS dataset [41] met our inclusion criteria for publicly available training datasets, except that it was assayed using the DASL microarray platform for FFPE specimens. The eight training datasets (including NHS/HPFS) have a total of 9336 overlapping genes. These genes form the basis of our analyses.

### Gene expression processing in curatedCRCData

curatedCRCData acquires and processes expression and clinical data from GEO [42] and The Cancer Genome Atlas [5] using the same pipeline as described for curatedOvarianData [43]. Briefly, raw data from Affymetrix platforms, if available, were pre-processed by either frozen Robust Multi-array Analysis [44] (U133a or U133 Plus 2.0 platforms U133a or U133 Plus 2.0 platforms) or Robust Multi-array Average [45] (others); otherwise pre-processed data as provided by the authors were used. Up-to-date maps from probe set identifiers to gene symbols were obtained, in order and according to availability, from BioMart [46], by BLAST of probeset identifiers, or from annotation files originally provided with the study submission. Genes with multiple probe sets were represented by the probe set with the highest mean across all.

### Evaluation of previously published discrete CRC subtypes

We used the “single subtype” classifiers provided by the Consortium (CMS) authors [10] and intrinsic subtype (CRIS) authors [11] to assign tumors in our 18 transcriptome datasets to the four CMS subtypes and the five CRIS subtypes, respectively. Expression values were per-gene median-centered first within each study before subjected to class assignment. For CMS, samples with posterior probability < 0.5 for all four subtypes are marked as “not labeled,” consistent as in [10]. We performed PCA [47] on the classifier genes in each dataset and used the first two PCs to visualize the major transcriptome shifts within the dataset. Similarly, silhouette widths based on different dissimilarity measures of the assignments in each dataset were calculated based solely on the expression of the classifier genes.

Silhouette width is a widely adopted clustering strength evaluation metric [17], aimed to quantify the level of “separateness” between a given class assignment within a dataset. For a given dataset with per-subject class assignments and a corresponding between-subject dissimilarity matrix, the silhouette width for subject *i*, *s*(*i*), is defined as follows. Let *a*(*i*) be the average dissimilarity between *i* and all other subjects assigned to the same class, and let *b*(*i*) be the maximal dissimilarity between *i* and any subject assigned to a different class, then

$$ s(i)=\frac{b(i)-a(i)}{\max \left\{a(i),b(i)\right\}} $$

The average of this index across samples can be the used as a quantitative metric for the level of separation between classes, with established and accepted thresholds [48]. In our analysis, i.e. for Fig. 2 and Additional file 1: Figure S1, the dissimilarity measure is calculated based on the signature genes used to define the CMS and CRIS subtypes (693 genes for CMS, 565 for CRIS). This is motivated by the notion that if CRC transcriptome is discrete, such discreteness should be observable at least on the genes used as signatures for such subtypes. Our selected dissimilarity measures include both parametric (Euclidean, Manhattan, 1 – Pearson correlation) and non-parametric (1 – Spearman correlation) measures.

In addition to classification validation, we also performed unsupervised clustering with k-medoids algorithm (paired with Euclidean distance) [20], non-negative matrix factorization [21], or consensus hierarchical clustering (paired with 1 – Pearson dissimilarity) [22] on the top 3000 genes with the highest variance (accounting for median 75% variability), or the CRIS signature genes (filtering for stromal contribution) in these datasets (Additional file 1: Figure S4). Clustering strength was evaluated with silhouette width, gap statistic [18], and prediction strength [19], to make sure results are not sensitive to any specific measurement. The unsupervised clustering analysis was to show that the observed lack of separation was an intrinsic feature of CRC transcriptome and not because we were applying the classifier trained from one dataset to potentially different studies.

### Continuous subtype discovery

We aimed to identify subtypes in CRC gene expression levels that were consistently present across different studies, without the a priori assumption that populations of different subtypes would be distinctly separable from each other. We approached this by performing PCA on the training datasets and constructing a network of correlated PCs. The procedure entails the following steps:

1. We performed PCA on each of the eight training datasets. Expressions are centered and scaled on a per-gene level first, per usual PCA standard. For each set, the loading vectors for the top 20 PCs were recorded. The number of PCs per dataset recorded and used in the following network analysis is large enough so that they are representative of the variability in each study (median percentage of total variability represented 59.08%, Additional file 2: Table S1).
2. For each pair of PCs from two different datasets, we calculated the Pearson correlation, denoted by *r*, of their corresponding loading vectors. That is, a total of ($20\times 20\times \frac{8\times 7}{2}$) Pearson correlations were calculated. Only common genes between the two datasets were used when calculating the correlations. We defined the PCs to be correlated to each other if |*r*| > 0.5. Related PCs were viewed as realizations of the same set of subtypes based on the PC scores. Note that PCs from the same datasets would never be related, since their loading vectors are orthogonal to each other (have a Pearson correlation of 0).
3. A network was constructed by placing edges between correlated PCs. We adopted a fast, greedy Girvan-Newman algorithm [49] as implemented in Cytoscape [50] to identify clusters of nodes (i.e. loadings) that are densely connected together by edges, and thus similar to each other, in this network.

For each large cluster in this network, the loading vectors of PCs within that cluster are similar to each other. The consensus of these loading vectors, defined through the average was used to assign scores based on each sample’s transcriptional profile (Additional file 3: Table S2). Specifically, the signs of each loading vector within a cluster was corrected for before taking the average, so that all of the loading vectors had positive correlations. The scores assigned by these consensus loadings were used as continuous subtype scores for CRC tumor characterization. See Additional file 1: Figure S5 for a detailed pipeline of the steps carried out for the identification of subtype scores.

### Validation of continuous subtype scores

To examine the external validity of the average loading vectors, we performed PCA on the 10 validation datasets and recorded the top eight loading vectors of each study. An average loading vector was considered as observed in, or correlated with, a validation dataset if it correlates with at least one of the eight top PC loadings with |*r*| > 0.5, the same standard as in the training process since the lowest ranking PC in the four clusters was PC8. With this definition we considered an average loading vector as validated if it was observed in at least 9/10 validation datasets.

The two particularly well-validated average loading vectors, those of clusters 1 and 2, were also examined against top PC loadings from normal tissue datasets, PCs randomly selected from the top 20 PCs of all eight training datasets, and permuted datasets, as negative controls. The normal tissue datasets were formed by limiting samples to three datasets with at least nine adjacent normal tissues available (GSE21510, GSE21815, TCGA.RNASeqV2) and only to those samples. The permuted datasets were formed by independently permuting the expressions for each gene in GSE13294. Since the permutation is random the selection of dataset does not affect our results; GSE13294 was chosen here because it had the median sample size across all 18 datasets.

### Calculation of continuous scores using average loading vectors

We used each average loading derived from the previous step to assign continuous scores to tumors based on their entire transcriptional profile. Suppose *x*_*j*_ denotes the gene expressions of the *j*-th tumor and *w*_*k*_ is the average loading vector of the *k*-th cluster, the score assigned for cluster *k* is then

$$ {s}_{jk}={\overset{\sim }{w}}_k^{\prime }{s}_j $$

where ${\overset{\sim }{w}}_k$ consists of the entries of *w*_*k*_ that correspond to genes present both in *w*_*k*_ and in sample *j*. ${\overset{\sim }{w}}_k$ is scaled so that ${\left\Vert {\overset{\sim }{w}}_k\right\Vert}_2=1$, just as with regular loading vectors. This essentially ensured that the assigned scores were on similar scales. Four such scores could be assigned, each from one of the identified four clusters. We name the two best-validated continuous scores as PCSS1 and PCSS2. Once calculated, the scores are further centered and scaled to mean zero and standard error one per study so that they are more comparable in a meta-analysis setting.

For assignment of continuous scores in the xenograft study GSE76402 and the CRC cell line dataset GSE59857, only the subset of 565 stroma-filtered genes from [11] were used.

### Generation of “signature” gene subsets for continuous scores

For PCSS1 and PCSS2, we calculated “pseudo-scores,” using only the genes with the largest absolute average loadings. Varying the number of top genes included, we compared the pseudo-scores with the original scores, and found that the pseudo scores reached Pearson correlations of > 0.9 with the original scores, even when only the top 200 genes were used (Additional file 1: Figure S7). In Additional file 3: Table S2, these are the top 200 genes ranked by absolute value of PCSS1 and PCSS2, respectively. In practice, any size of signature gene subset can be chosen by setting an ideal correlation cutoff.

### Fixed effects model on correlations between continuous scores and clinical metadata

Individual univariate logistic regressions were fitted for the binary variables (individual subtypes, MSI, location, stage, grade) on each dataset. That is, the clinical variables were used as outcomes and continuous scores predictors. Firth’s penalized likelihood [51] was used in cases where perfect separation or single-level outcomes occurred to obtain estimates convergence. Log odds ratios from different studies were then pooled together with fixed effects models [13] to give estimates for overall effects and *p* values. For survival analyses, Cox proportional hazard models on DFS were fitted for each dataset before the log hazard ratios were pooled together with the fixed effects model.

### Comparison between continuous scores and CMS/CRIS subtypes in characterizing molecular, histopathological, and clinical variables

We fit logistic and Cox regression models with MSI status, location, stage, grade, or DFS as the outcome variable. For each outcome variable, three different sets of covariates were used: (1) PCSS1 and PCSS2 scores; (2) CMS subtypes; and (3) both the continuous scores and discrete subtypes. LRTs were performed comparing models 1 vs. 3 and 2 vs. 3. The AIC from fitting each model and *p* values from LRT are used to assess the capability of continuous scores and discrete subtypes in characterizing outcome variables. Significant *p* values from LRT would indicate that the reduced (i.e. with continuous scores or discrete subtypes only) model is not sufficient in replacing the full (i.e. with scores and discrete subtypes) model. Similarly, a model with higher AIC indicates it is performing worse in fitting the data. Our results suggest that the continuous scores are preferable than discrete subtypes for characterizing outcome variables by both criteria.

### Gene set enrichment analysis

Ranked gene set enrichment analyses were performed on both scores to find pathways that hit the top genes more often. Pathway gene sets are obtained from BioCarta. Mean-rank gene set enrichment analysis [52] was performed, which test for the whether the pathway gene sets are more highly ranked in terms of the continuous score loadings compared to randomly chosen genes.

## Additional files

Additional file 1: All supplemental figures. (PDF 5182 kb)

Additional file 2: **Table S1.** Variance and percentage variance explained by the top 20 PCs in all 18 datasets. (XLSX 13 kb)

Additional file 3: **Table S2.** Average loading vectors used to assign continuous subtype scores. For each continuous score, the top genes ranked by the absolute loadings are most representative and can be used as signatures to reproduce the score (Additional file 1: Figure S7). (XLSX 681 kb)

Additional file 4: **Table S3.** PCSS1 and PCSS2 are validated in 9 of the 10 validation datasets. A subtype score is considered to be validated in a dataset if its average loading is correlated with any of the top eight PC loadings of the dataset with Pearson correlation > 0.5 (see “Methods”). (XLSX 9 kb)

Additional file 5: **Table S4.** Statistics from fitting regression models with subtype (scores) to outcome variables (MSI, stage, grade, location, and DFS). (XLSX 23 kb)

Additional file 6: **Table S5.** Biocarta pathways that are enriched (Bonferroni corrected *p* < 0.05) for the genes with large weights for PCSS1 and PCSS2. The subtype scores thus characterize the variations in the enriched pathways. (XLSX 9 kb)

Additional file 7: **Table S6.** Meta-analysis of comparing continuous scores with CRIS subtypes. (XLSX 20 kb)
