# Supplementary material for: Continuity of transcriptomes among colorectal cancer subtypes based on meta-analysis
Source: Genome Biol. 2018 Sep 25;19:142. doi: 10.1186/s13059-018-1511-4 (PMC6154428; doi:10.1186/s13059-018-1511-4)
Supplement: Supplementary file 1 — All supplemental figures. (PDF 5182 kb) [file 13059_2018_1511_MOESM1_ESM.pdf]

Fig. S1: **Average silhouette width indicates no separation between the transcriptomes of the CMS and CRIS subtypes, when evaluated across different dissimilarity metrics. A)** Average silhouette widths indicate that there is no separation between the CRIS subtypes in our 18 datasets, as well as the additional CRC xenograft dataset (GSE76402) and CRC cell line dataset (GSE59857). Dissimilarity is calculated using the stroma free 565 CIRS signature genes in [1]. This panel is the CRIS equivalent of Figure 2A. **B)** The lack of separation between subtypes is consistent when evaluated using different dissimilarity metrics for both CMS and CRIS subtypes.

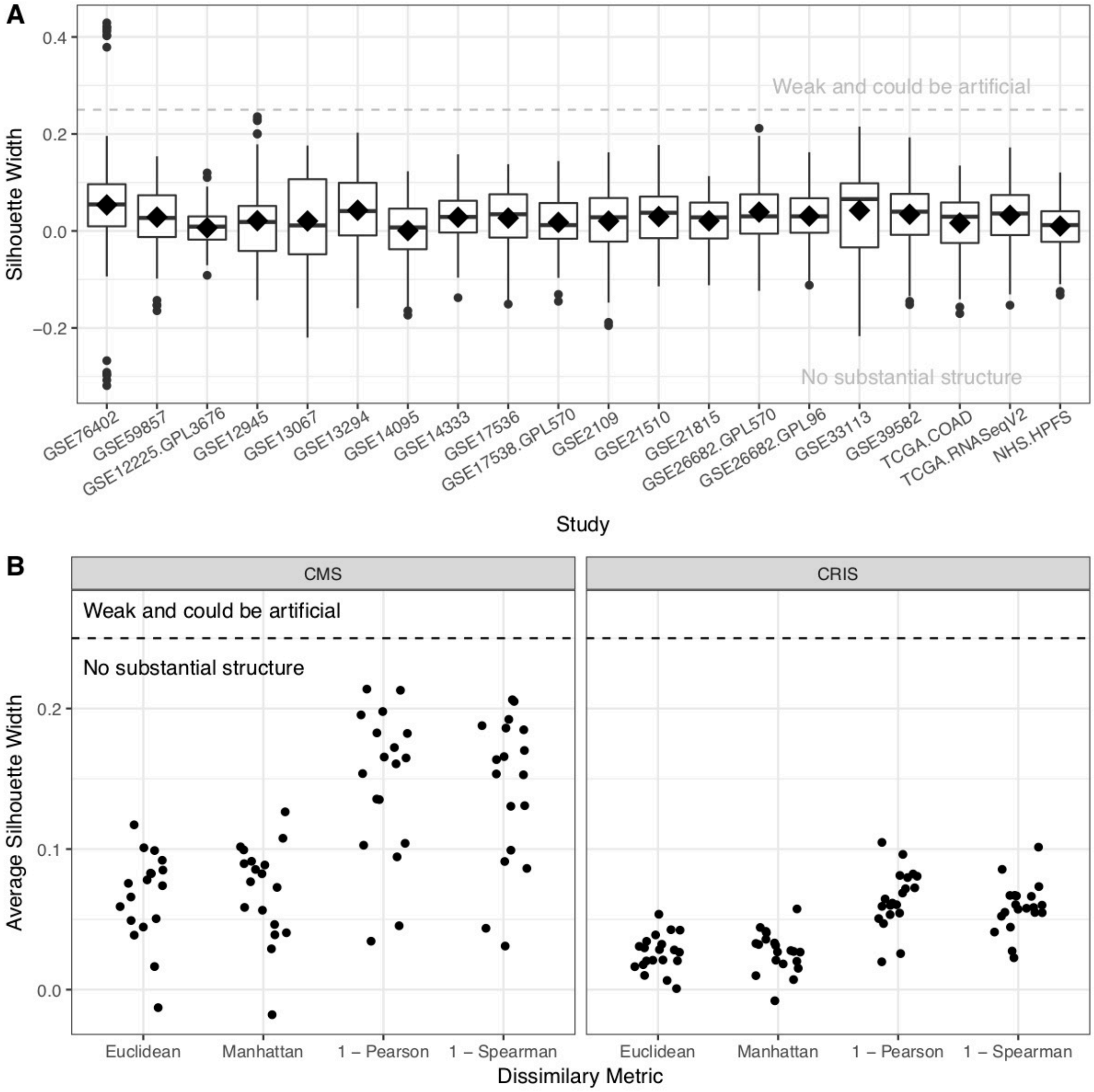

Fig. S2: Subtypes reported by the Consortium visually show little to no separation in the top four PCs in all 18 datasets.

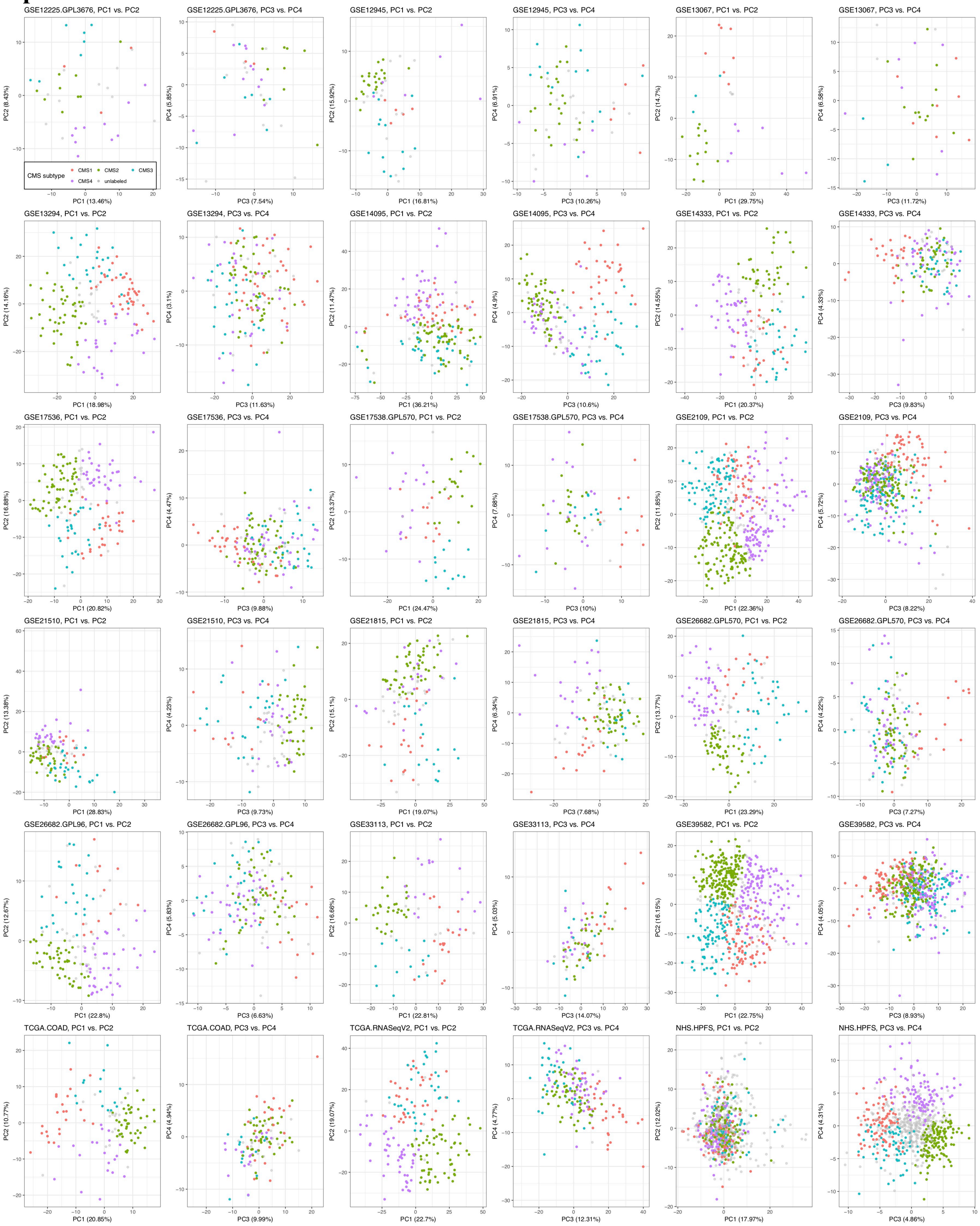

Fig. S3: Breast cancer patients' transcriptome visually form distinct subtypes (clusters) in the first four PCs with respect to subtype assignment [2]. This is in contrast against CRC patients.

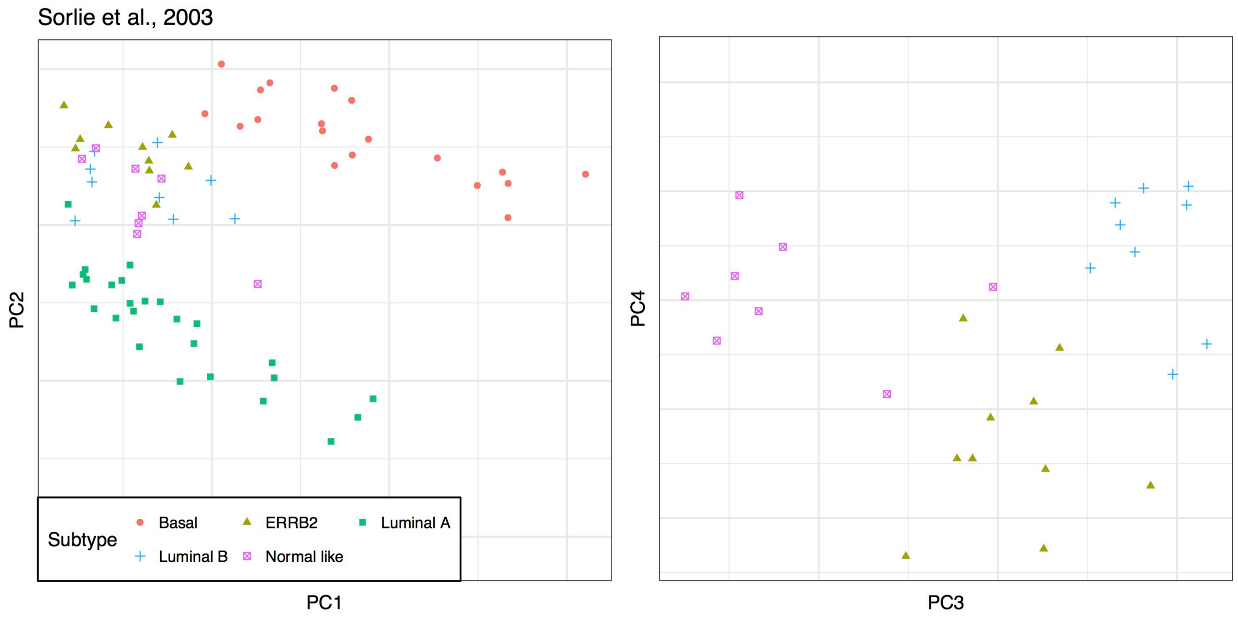

Fig. S4: **Examination of transcriptome discreteness on datasets with unsupervised clustering.** Measure of discreteness (y-axis) was evaluated on the top 3000 most variable genes (green), or the 565 CRIS signature genes (red), across different number of clusters (x-axis). Prediction strength, gap statistic, and average silhouette width are evaluated for each dataset when clustered to 2-8 clusters. For gap statistic and average silhouette width, results from k-medoid clustering was evaluated; for average silhouette width, k-medoid, non-negative matrix factorization, and consensus hierarchical clustering were evaluated. Strong evidence for discrete classes would be supported by the peaking of the statistics at a particular number of clusters (especially, given the CMS and CRIS subtype claims, at 4 or 5 clusters), which was absent in these results. Additional notes: 1) results for CRIS signature genes also include two additional CRC xenograft and cell line datasets; 2) difference in gap statistic is mostly driven by sample sizes; the larger the number of samples clustered, the higher the statistic is. Error bars indicate standard errors.

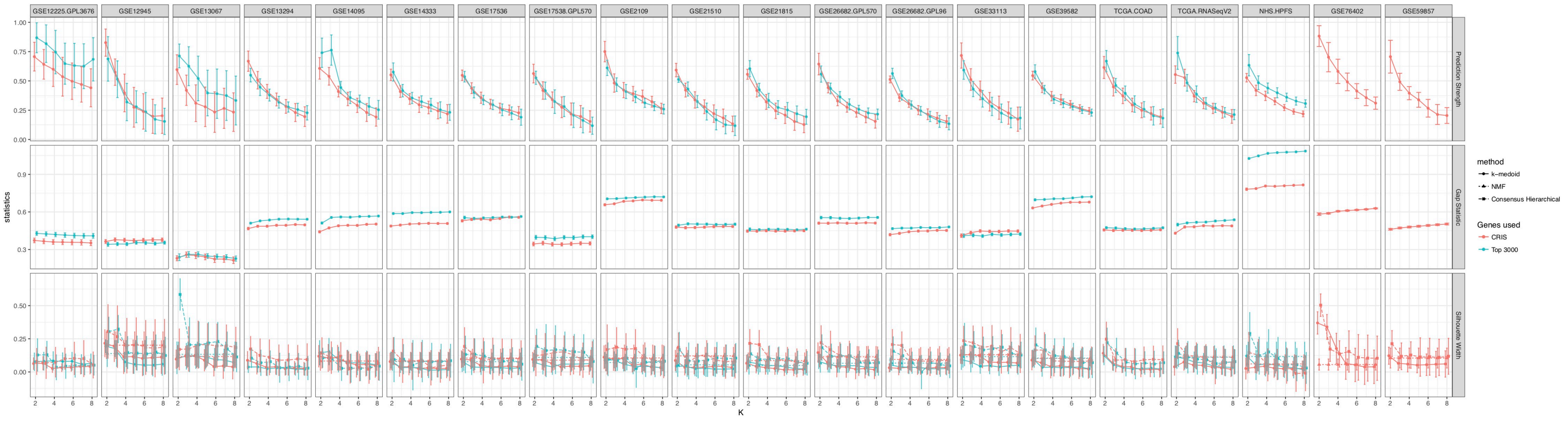

Fig. S5: Steps carried out in this study for the identification of continuous subtype scores for CRC.

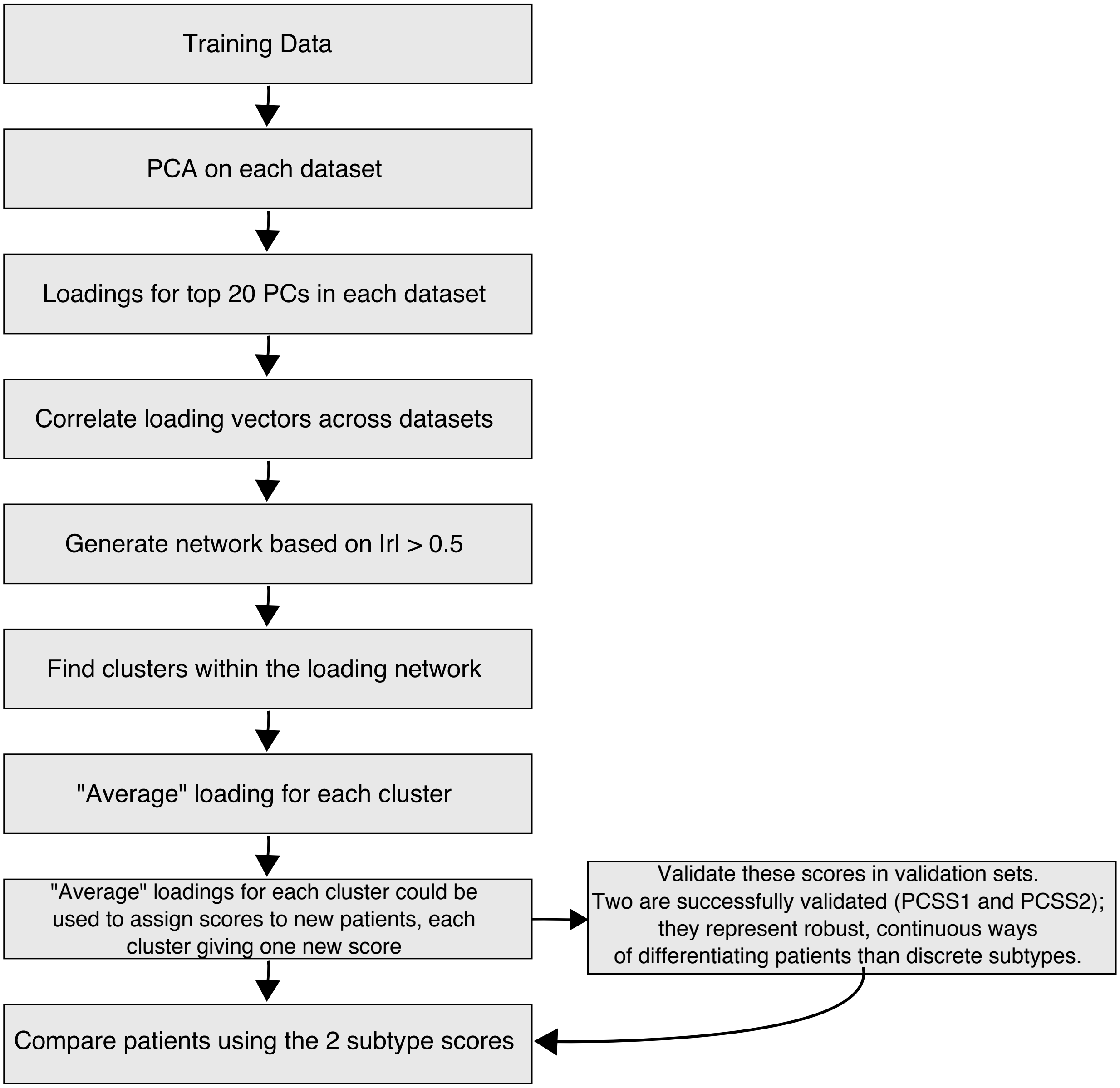

Fig. S6: PCSS1 and PCSS2 are validated in both training and validation datasets, but not in datasets with normal tissues only, randomly selected PCs, or "randomized" datasets obtained by permuting expressions for each gene. The validation of each continuous score in a dataset is quantitatively represented by the highest absolute correlation between that score's average loading vector and those for the top eight PCs of the dataset (**Methods: Validation of continuous subtype scores**). The permuted datasets have the weakest correlations with PCSS1 and PCSS2 because they were derived by permuting expression values for each gene independently, thus breaking any structures there might be in the transcriptome.

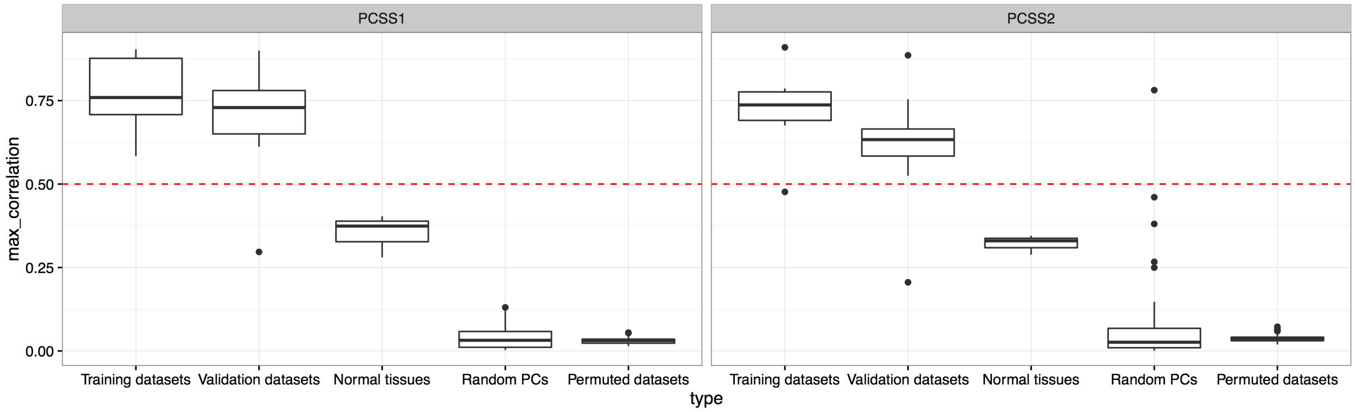

Fig. S7: Pearson correlations of "pseudo"-continuous scores with PCSS1 and PCSS2 exceed 0.9 even with only the top ~200 genes with high absolute loadings, suggesting in practice the top genes can be used as signatures to assign continuous scores in place of the transcriptome. Error bars indicate 95% confidence intervals. The correlations were taken average across the entire 18 data sets.

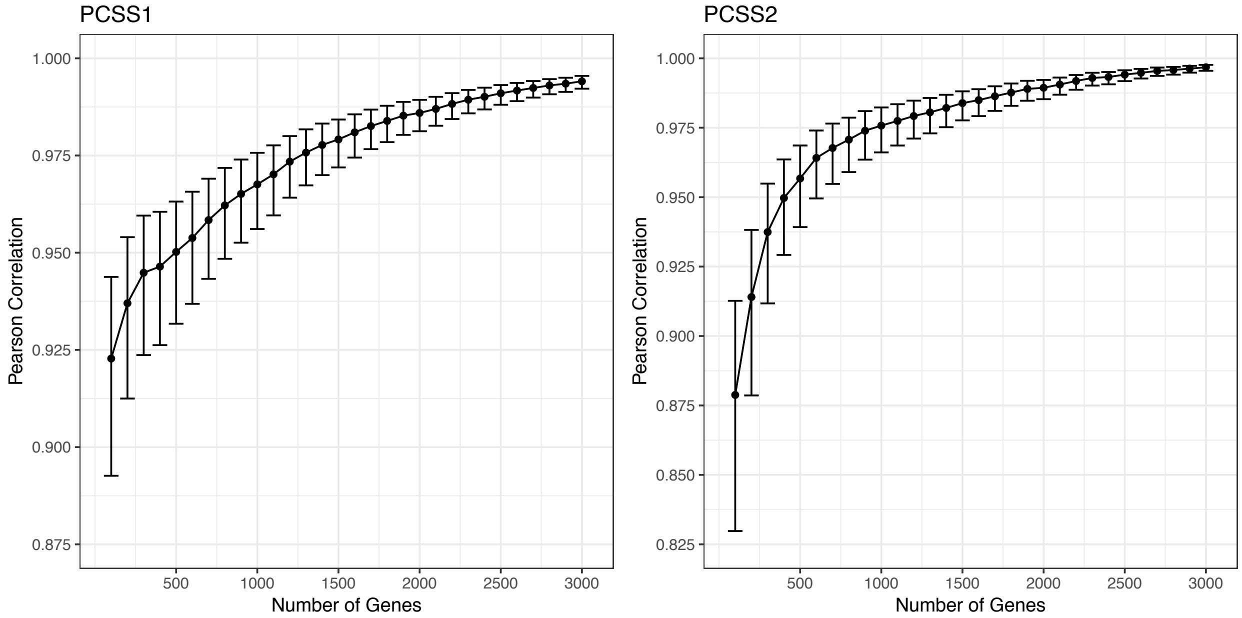

Fig. S8: Transcriptional profiles of MSI gene signatures corresponds strongly to MSI status, but do not cluster discretely. A) PCSS1 and PCSS2 form a continuous space that strongly correlate with, but do not separate MSI status. B) Silhouette width provides no evidence for discrete separation of the transcriptional profiles of MSI and MSS patients on a MSI gene signature transcriptomes, as provided in [3]. Boxplots show the distribution of silhouette widths of all samples in each dataset, whereas diamonds mark out average silhouette widths.

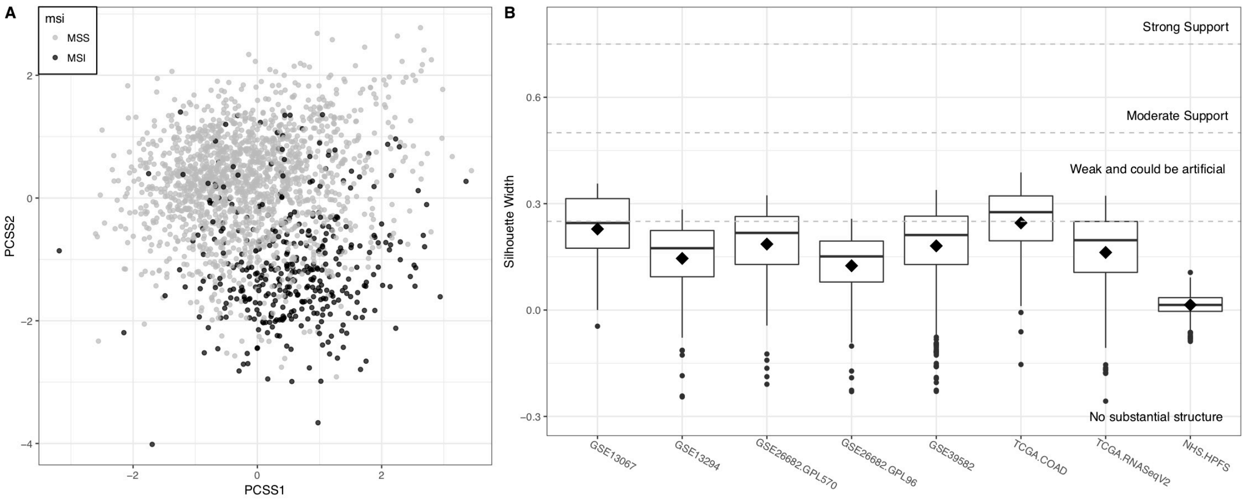

Fig. S9: PCSS1 and PCSS2 further differentiate prognostic in high-risk CMS4 subtype. **A)** For CMS4 patients, those with high PCSS1 or PCSS2 (defined as either score being greater than its upper-quartile) have significantly worse survival through meta-analysis Points and error bars in the figure indicate log hazard ratios and confidence intervals within individual study, as well as the aggregated results from meta-analysis ("RE Model"). **B-C)** Independently, high PCSS1 (PCSS1 greater than upper-quartile) and high PCSS2 (PCSS2 greater than upper-quartile) are also associated with worse prognosis in CMS4 patients. **D)** For CRIS-B patients, high PCSS1 (PCSS1 greater than median) is associated with worse survival, but the effect is not statistically significant.

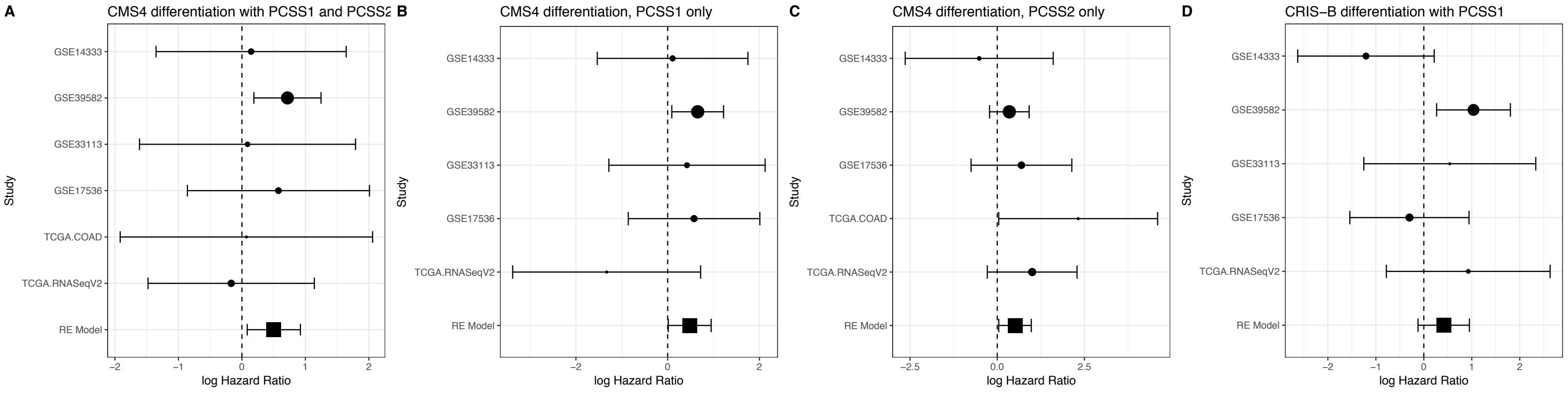

Fig. S10: Correlations between the high risk discrete CRIS-B subtype and continuous scores are consistent across studies and stromal content. The log odds-ratios of regressing CRIS-B subtype on PCSS1 (left) and PCSS2 (right) across studies are visualized as forest plots. The effect sizes are consistent across all 18 bulk tissue studies, and importantly, also across the xenograft dataset (GSE76402) and CRC cell line dataset (GSE59857). The other CRIS subtypes also have consistent correlations with PCSS1/2 (**Supplemental Table 6**).

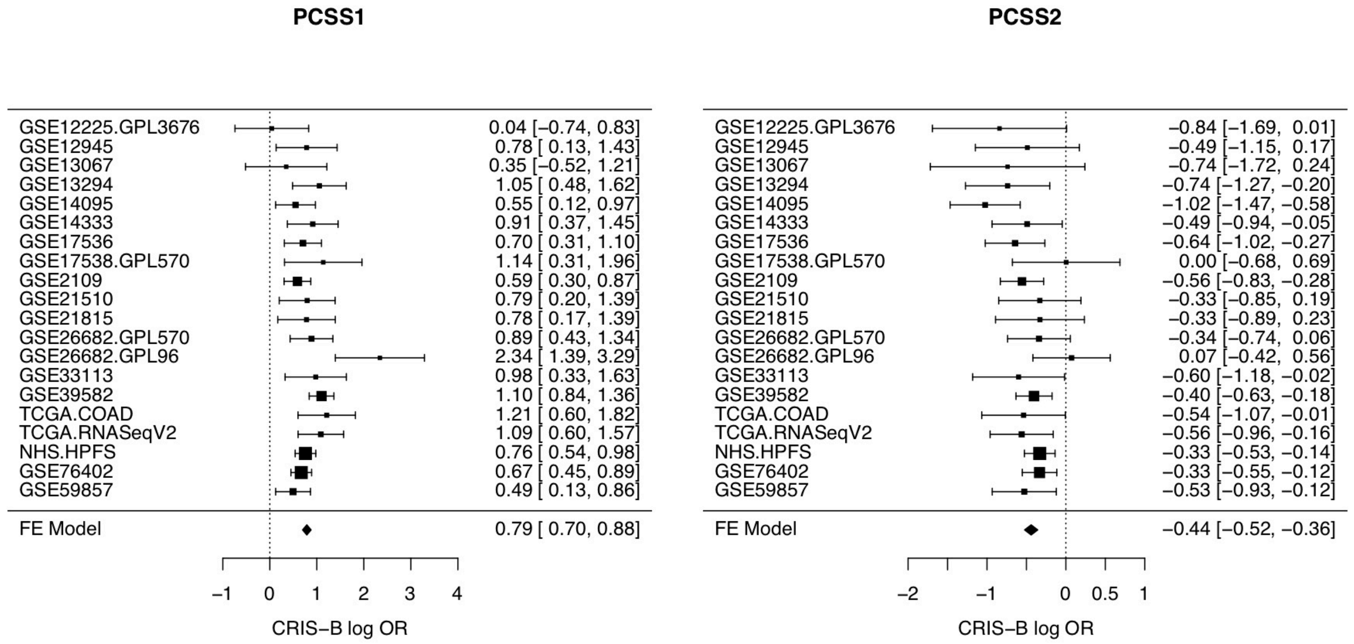

Fig. S11: Same as for CMS, continuous scores are more closely associated with molecular and clinical/pathological variables than CRIS subtypes. This figure is the CRIS equivalent to the results included in **Figure 4C**. Likelihood ratio tests were used to compare the full model, containing both CRIS subtype and score as predictors, to a simplified model containing only CRIS (left) or score (right) as predictor. Test results for different datasets (p-values) are represented by points in the box plots. Interpretations for p-values are the same as in **Figure 4**.

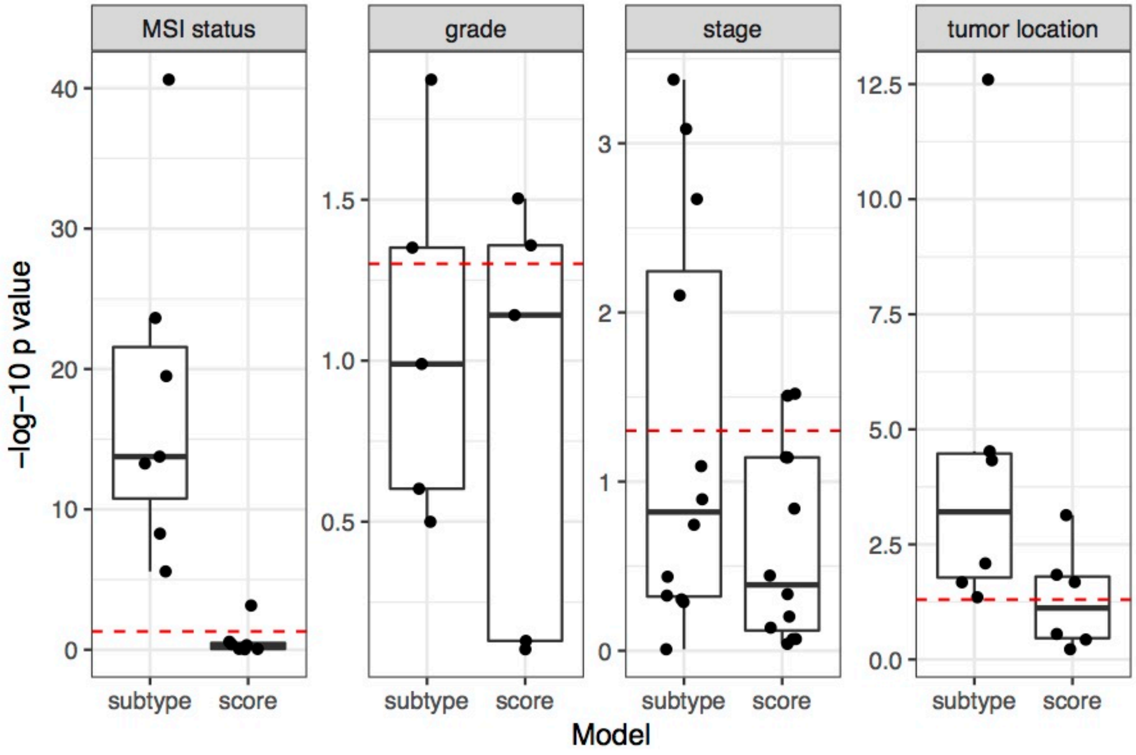

Fig. S12: PCSS1 and PCSS2 are distributed unimodally (panel A), and visually don't separate the CMS subtypes (B), as quantitatively evidenced by silhouette width (C). A) 2-dimensional density contours show that the joint distribution of PCSS1 and PCSS2 is unimodal. **B)** PCSS1 and PCSS2 visually don't separate CMS subtypes. **C)** Average silhouette widths on PCSS1&2 (calculated using Euclidean distance) indicate no separation between the subtypes. Error bars indicate standard errors.

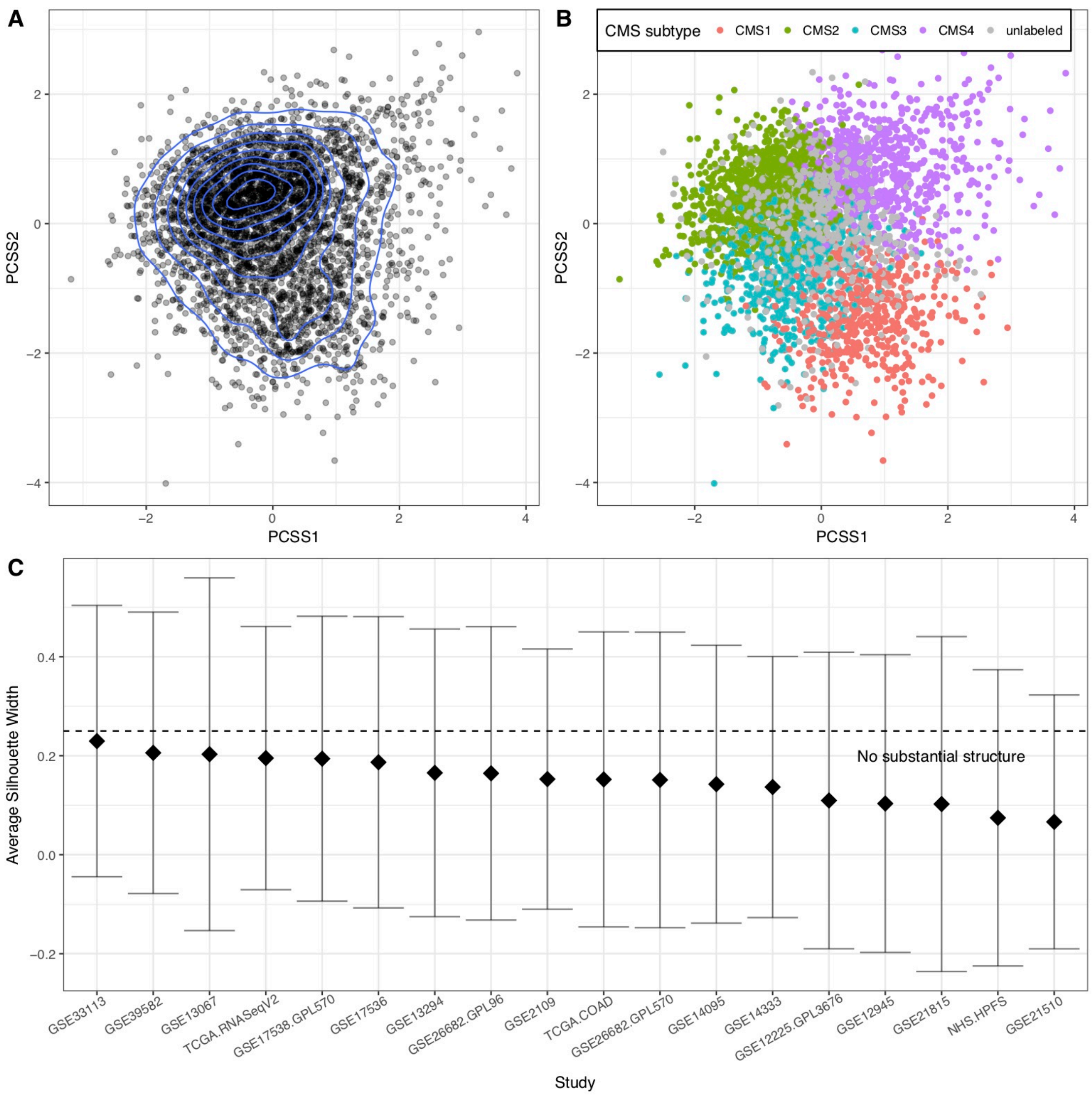

References

1. Isella C, Brundu F, Bellomo SE, Galimi F, Zanella E, Porporato R, Petti C, Fiori A, Orzan F, Senetta R, et al: **Selective analysis of cancer-cell intrinsic transcriptional traits defines novel clinically relevant subtypes of colorectal cancer.** *Nat Commun* 2017, **8:**15107.
2. Sorlie T, Tibshirani R, Parker J, Hastie T, Marron JS, Nobel A, Deng S, Johnsen H, Pesich R, Geisler S, et al: **Repeated observation of breast tumor subtypes in independent gene expression data sets.** *Proc Natl Acad Sci U S A* 2003, **100:**8418-8423.
3. Jorissen RN, Lipton L, Gibbs P, Chapman M, Desai J, Jones IT, Yeatman TJ, East P, Tomlinson IP, Verspaget HW, et al: **DNA copy-number alterations underlie gene expression differences between microsatellite stable and unstable colorectal cancers.** *Clin Cancer Res* 2008, **14:**8061-8069.
